# Consciousness, Cognition and the Cognitive Apparatus in the Vedānta Tradition

**DOI:** 10.4103/0973-1229.77427

**Published:** 2011

**Authors:** R. Balasubramanian

**Affiliations:** * *Chairman, Indian Philosophical Congress. President, Afro-Asian Philosophy Association*

**Keywords:** *Consciousness*, *Self*, *Vedānta tradition*, *Mind*, *Self*, *Intentionality*, *Sartre*, *Śankara*, *Popper*, *Husserl*, *Enworlded subjectivity*

## Abstract

A human being is a complex entity consisting of the Self (also known as Consciousness), mind, senses and the body. The Vedānta tradition holds that the mind, the senses and the body are essentially different from the Self or Consciousness. It is through consciousness that we are able to know the things of the world, making use of the medium of the mind and the senses. Furthermore, the mind, though material, is able to reveal things, borrowing the light from consciousness. From the phenomenological point of view, we have to answer the following questions: how does one know the mind/the mental operations/the cogitations of the mind? Does the mind know itself? Is it possible? There is, again, the problem of the intentionality of consciousness. Is consciousness intentional? According to Vedānta, consciousness by its very nature is not intentional, but it becomes intentional through the mind. The mind or the ego is not part of the consciousness; on the contrary, it is transcendent to consciousness. It is difficult to spell out the relation between consciousness and the mind. How does consciousness, which is totally different from the mind, get related to the mind in such a way that it makes the latter capable of comprehending the things of the world? The Vedānta tradition provides the answer to this question in terms of the knower-known relation. Consciousness is pure light, self-luminous by its very nature, that is, although it reveals other objects, it is not revealed by anything else. When Sartre describes it as nothingness, bereft of even ego, it is to show that it is pure light revealing objects outside it.

## Basic Problem

The concept of the enworlded subjectivity is problematic as it involves two notions, worldliness or embodiedness on one hand, and subjectivity on the other, which do not go together and create a tension in our understanding. Consciousness is the subjectivity; having no relation with any object, it is transempirical, transrelational, and therefore, disembodied. It means that there is a dichotomy between consciousness (subjectivity) and the world of objects presented to it. However, there is the involvement of consciousness in the objects of the world, that is, consciousness becomes worldly. The important question that we have to ask is: “How is it possible that consciousness, which is essentially different from everything else presented to it as its object, gets itself involved in the objects of the world surrounding it, losing its identity in such a way that it is not even reckoned as an entity in its own right along with other objects?” This is the problem of the enworlded subjectivity. At the commencement of his commentary on the *Brahma-sūtra*, Śa
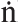
kara draws our attention to this problem of the enworlded subjectivity. The dichotomy between consciousness and what is presented to consciousness shows that the latter is “transcendent” to it and is, therefore, an *object* of consciousness, whereas consciousness which reveals whatever is presented to it is the *subject*. The distinction between consciousness and what is presented to consciousness is what Śa
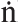
kara calls the distinction between “*asmad*” and “*yuśmad*”, the subject and the object, the Self and the not-Self. Absorbed as we are in the transactional world, we fail to notice the radical distinction between the subject and the object, and confuse and mix up the one with the other. The naive and natural mode of thinking and its resultant activity are due to our ignorance of the Self, the pure consciousness, the transcendental subjectivity. What Husserl calls the life-world, the lived experience (*Lebenswelt*), is spoken of as *loka-vyavahāra* by Śa
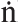
kara. One could notice the natural attitude fully manifested in the activities-cognitive, affective and conative-of our daily life.^1^

## Foundational Consciousness and Functional Consciousness

According to Advaita, the inquiry that is needed for unravelling the mystery of the enworlded subjectivity is facilitated by the principle of consciousness; in other words, the inquiry is phenomenologically grounded. Advaita holds that the evidence of consciousness is apodictic. If we say that something is such-and-such and that something else is not such-and-such, it is on the basis of the evidence of consciousness. How the principle of consciousness regulates our knowledge claims can be formulated in two ways, positively as well as negatively. What is affirmed by consciousness through its intentional performance cannot be denied; and what is denied by consciousness through its intentional performance cannot be affirmed. There is no other principle for the acceptance or denial of anything. Consciousness by its very nature is revelatory. It reveals the mental states and the cogitations of the mind on its own; it also reveals the external objects through the mind and the senses. According to Advaita, though consciousness by its very nature is not intentional, it nevertheless becomes intentional through the functioning of the mind. It is in this connection that we have to make the distinction between foundational consciousness and functional consciousness. While the former is non-relational, the latter is relational. It is the association with the mind that makes it relational, and so we speak of the intentionality of consciousness.

The empirical journey of the transcendental subjectivity to the world horizon passes through the landmarks of the mind, the senses and the body. As it moves from its non-dual, non-relational state, there is progressive entanglement with the objects transcendent to it until it emerges as the enworlded subjectivity in all its completeness. We have seen that there is a need for a rigorous regressive analysis of the objects presented to consciousness for the purpose of catching hold of the inward self, the transcendental subjectivity, as the phenomenological residuum; and in this regressive analysis, the homecoming starts from the outer world to the inner world, and then from the inner world to the Self, which ultimately remains alone.

The internal organ (*anta*
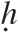
*kara*
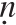
*a*), which is commonly called the mind, is the first entity with which consciousness comes into relation. The association of consciousness with the internal organ gives rise to the emergence of the “I” or ego. Though the ego or “I” is transcendent to it, and is, therefore, different from it, it nevertheless identifies itself and appears as “I.” In the words of *Sureśvara*, it is as though it has put on the mask of the “I.” It is this “I” that is commonly spoken of as the “empirical I,” as the “ego-consciousness,” as the “factual I”; it is the *epoche*-performing ego of Husserl. The relation between the transcendental Self and the empirical “I” is the relation of the revealer and the revealed (*avabhāsaka-avabhāsya-sambandha*). Once the ego or “I” emerges at this level, that is to say, once the transcendental Self puts on the mask of the “I,” then it begins to function through its intentional acts, taking advantage of the senses and the body. In this process, it identifies itself with the mind, with the senses and then with the body. This identification is evident in the claims that we make in our daily life such as “I am happy,” “I am blind,” “I am stout” and so on. Functioning through the mind-sense-body complex, the “I” comes into relation with the objects of the external world, develops pragmatic attitude toward them through its intentional acts, considers those objects which are helpful to it as good and those which are not helpful as bad and behaves as if it were helped or hindered by them. The transcendental subjectivity which provides meaning and validity to the objects is now thrown into the world as the embodied subjectivity as if it were an entity in need of sustenance from the very objects which are “constituted” or “accomplished” by it. Nothing is more tragic, more poignant, than this existential situation, in which the source becomes the supported, and the helper, the helped. Such is the entanglement of the self as the embodied subjectivity functioning as a being-in-the-world, overwhelmed by the natural attitude of “That I am” and “That is mine.” The journey of the Self is one of progressive “fall.” Sureśvara sums up, in a language which is terse, the emergence of the enworlded subjectivity as follows:

The internal organ, being delimited by the “I”-notion, becomes an object directly to the reflected consciousness (i.e., cidābhāsa), of which the immutable, inward Self is the cause. Now, except the relation of the revealer and the revealed, no other relation is tenable between the “I” and its knower. Appropriating the internal organ as its own and putting on the mask of the “I,” the Self becomes fit enough for the helped-helper relation, and comes to be related to the external objects, helpful or harmful as the case may be, claiming them its own.^2^

## Regressive Analysis

The phenomenological reflection through a rigorous regressive analysis helps us to remove the coverings of the Self, including the mask of the “I,” which are external to it, and know the Self as it is. If the transcendental subjectivity is the real Self (*mukhyātmā*), the “I,” the empirical self, which functions through the mind-sense-body complex, is the false self (*mithyātmā*).^3^ Since the “I” brings together the Self and the objects of the world, its role is crucial. So long as there is the “I,” there is the world; and when the “I” goes away, there is no more the familiar world horizon.

Advaita holds that the evidence of consciousness is the only evidence that is certain and apodictic for any claim that we make to the effect that we know something or that we do not know something. Every source of knowledge is dependent on consciousness; whatever be the source of knowledge, be it perception or inference or scripture, it presupposes consciousness as its ultimate source. That which is the presupposition of every kind of knowledge and every source of knowledge cannot be validated by any other principle. When we say that something is the case or that something is not the case, it is on the basis of the evidence of consciousness that we say so. In the language of William James, Advaita may be characterised as “radical empiricism.” Advaita maintains that whatever is shown or revealed by consciousness cannot be rejected, and that whatever is not shown or revealed by consciousness cannot be accepted. In fact, it goes to the extent of saying that even the claim that something is not known presupposes the evidence of consciousness, just as the claim that something is known presupposes the evidence of consciousness. There are two kinds of seeing or vision (*d*
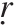

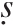

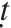
*i*), real (*pāramārthikī*) and actual (*laukikī*), according to *Śa*
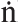
*kara*.^4^ The former is the seeing or vision of consciousness, while the latter is the seeing or vision of the mind, or through the mind. This distinction between the two kinds of seeing is of great significance, as it highlights the role of consciousness as the witnessing or the knowing principle. When I say, “This is a table,” and “That is a tree,” I have the knowledge of the object through the mind. It is what is called “modal cognition,” that is, cognition through the mode of the mind (*v*
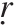
*tti-jñāna*). In these cases, consciousness reveals things through the mind; and in the absence of consciousness, mind by itself, which is material, cannot give us knowledge of anything. It is not the case that every case of seeing or knowing is through the mind. There are cases where consciousness without the medium of the mind or any other medium, directly reveals the object. The case of the ego or “I” is a standing example in this regard. We have already pointed out that the ego which is revealed by consciousness is transcendent to consciousness. Though the ego or “I” may appear to be the knower (*jñātā*) in respect of objects external to it, the real position is that it is an object in relation to consciousness. Advaita holds that the ego or “I” is directly revealed by consciousness (*kevala-sāksi-bhāsya*) without the intervention of the mind. Similarly, when I say, “I am ignorant of something,” “That is unknown to me,” it is on the basis of the direct evidence of the witnessing consciousness. In short, every kind of claim that we make-that something is *known*, or that something is *unknown*-is on the basis of the transcendental consciousness which reveals objects directly, or through the intentional performance of the mind. The evidence of the transcendental consciousness is intrinsically valid. Therefore, it is considered to be the principle of all principles, the source of all of our claims-claim to knowledge as well as claim to ignorance.

## Metaphysical Thesis

On the basis of the phenomenological method, Advaita maintains that whatever is cognised must be admitted to be existent. Every cognition has a cognitum. And this is as much true without regard to what is called erroneous cognition as it is true in respect of veridical cognition. In the well known example of the rope-snake error, a person first of all cognises the object in front as a snake and gives expression to his cognition by saying, “This is a snake.” Subsequently, on a closer view, he cognises it as a rope, corrects the mistake he has committed, and says, “This is not a snake, but a rope.” Although the initial cognition affirms the existence of a snake, the subsequent cognition, which sublates the earlier cognition, denies it by affirming the existence of the rope. Negation presupposes affirmation: that is to say, what is initially affirmed alone can be denied subsequently.^5^ The fact is that “snake” was presented to consciousness as an object, and it was cognised as such by the person concerned at that time, in that place. What is cognised cannot be dismissed as non-existent.^6^ At the same time, since the subsequent cognition has sublated it, it cannot be said to be existent.^7^ On the basis of the evidence of the intentional acts of consciousness, we have to say that the rope-snake has a peculiar ontological existence such that it can be characterised neither as non-existent nor as existent. Therefore, Advaita says that the rope-snake has to be accorded some kind of reality, what it calls phenomenal reality (*prātibhāsika-sattā*), in the world horizon. Advaita examines the objects of the external world such as the table and the tree by applying the same phenomenological method. These objects, like the rope-snake, are not only cognised, but also suffer sublation. Although they are affirmed by our waking experience, they are denied by our dream experience, just as what is affirmed by the dream experience is denied by the waking experience. What is seen in the daily waking experience gets sublated when someone is fortunate enough to realise the transcendental Self as the sole reality. As in the case of the rope-snake, the objects of the external world must be accorded some reality, since they have been cognised and sublated. Noticing the difference between an object of erroneous cognition and an object of normal waking consciousness,^8^ Advaita says that objects such as the table and the tree have empirical reality (*vyāvahārika-sattā*). The transcendental consciousness is not an “object” like the rope-snake or a tree which can be cognised. Since it is self-luminous, it is always known; or, as *Śa*
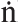
*kara* would put it, it does not remain unknown. There is no possibility of its sublation. What is other than consciousness is “object” which is dependent on consciousness for its meaning and validity, and so the question of sublation of consciousness by “object” does not arise. Consciousness, according to Advaita, possesses absolute reality (pāramārthika-sattā). The phenomenological method which Advaita pursues results in the theory of the levels of reality-what is phenomenally real, what is empirically real and what is absolutely real. The transcendental consciousness, which is autonomous and absolutely real, is one and non-dual. There is nothing else, similar or dissimilar to it, which is autonomous. It is homogeneous and indivisible. It can neither be seen, nor can it be sublated. Therefore, it is unique. Its nature being what it is, Advaita, following the Upani
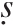
adic lead, characterises it as “one only without a second” (*ekameva advitīyam*).^9^ The rigorous pursuit of transcendental phenomenology to its logical end consummates in the metaphysics of non-dualism to which Advaita is committed.

## The Three Worlds and Beyond

There are at least two models of the three worlds. One is the Upani
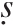
adic model which speaks of the world of waking experience (*jāgrat*), the world of dream experience (*svapna*) and the world of deep sleep experience (*su*
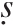
*upti*). Every normal human being not only experiences, but also is aware of these three worlds. The *Upani*
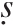
*adic* tradition also mentions the fourth (*caturtha*) as what is beyond these three worlds, and gives us the assurance that it is possible for everyone to experience the fourth, the beyond, by transcending the three worlds. In recent times, Karl Popper, and following him, John Eccles, spoke of three worlds constituting the whole reality. They call them World 1, World 2 and World 3.^10^ They do not, however, speak of what is beyond these three worlds.^11^ Let us first consider the Upani
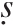
adic model.

## The Upanisadic Model of Three Worlds

Advaita may be characterised as radical empiricism as it examines every aspect of our experience at all levels-waking, dream and sleep-for the purpose of ascertaining the nature of consciousness. The three states of experience constitute the three worlds in which all the *jīvas* live and move about. Unlike other animals, the human being not only experiences the three worlds, but is also aware of these worlds and knows the similarities and differences among them. The foundational consciousness which is present in all of them becomes functional through its association with the mind, the senses and the body, which serve as the media for its functioning. When it is thus associated with the mind-sense-body complex, it becomes functional, relational and manifold. It may be pointed out in this connection that in the Vedānta tradition, the two words “consciousness” (*cit*) and “experience” (*anubhava*) are used as synonyms both in the absolute and the relative sense. When we speak of the foundational consciousness as one and non-dual, we use the term “consciousness” in the absolute sense, whereas when we refer to functional consciousness, we use it in the relational sense. What is really one appears to be many because of the objects with which it is related. The same is the case with “experience,” which can be used both in the absolute and the relative sense.

The analysis of the triple stream of experience of the *jīva* helps us to understand the concept of the Self-in-the-body, that is, consciousness in its embodiment, both epistemically and metaphysically. Though consciousness by its very nature is not intentional, it becomes intentional through the mind which plays an important role in the states of waking and dream. The following diagram contains the salient features such as the contextual names and conditioning factors of the functional consciousness in the triple stream of experience (*jīvaavasthā-traya*jīva) [Table 1].

**Table 1:** Triple stream of experience (*avasthā-traya*)

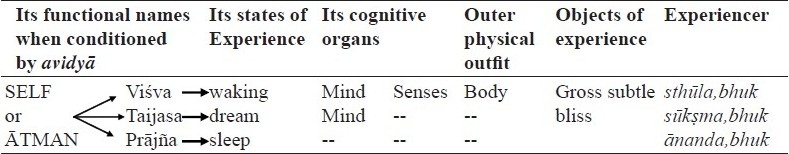

## Intentionality of Consciousness

Let us first consider the epistemological problem of the intentionality of consciousness. The theory of the intentionality of consciousness which plays an important part not only in the phenomenology of Husserl, but also in the phenomenological ontology of Sartre deserves careful consideration.^12^ Husserl assigns to consciousness not only an important place in his system, but also makes it the starting point of philosophical investigation. Sartre does not disagree with Husserl on this issue, though he was thoroughly unhappy with the latter’s formula of “turn to the subject,” which replaced the earlier formula of “turn to the object.” To both Husserl and Sartre, consciousness is intentional. However, the theory of intentionality of consciousness takes a new dimension in the Sartrean phenomenological ontology, for, Sartre, unlike Husserl, rejects the transcendental “I,” but clings to the intentional consciousness and the intended objects, and makes consciousness a non-substantial and impersonal being, a “free spontaneity,” a “great emptiness,” a “wind blowing towards objects.”^13^ Consciousness, Sartre says, is always consciousness *of* something. It is always pointing toward that which is beyond it. There is no consciousness, according to Sartre, which is not related to a transcendent object.^14^ Following Husserl, Sartre maintains that intentionality is essential to consciousness; consciousness, that is to say, is defined by intentionality. He considers this to be “the fruitful definition”^15^ of consciousness. Sartre thus accepts Husserl’s theory of the intentionality of consciousness. There is, however, an important difference between Husserl and Sartre even here; although for Husserl, intentionality is *one* essential feature of consciousness, for Sartre, intentionality is consciousness. For the present, we can ignore this difference between them, as it does not in any way affect the problem of the intentionality of consciousness which we are now considering.

Advaita *Vedānta*, which is transcendental phenomenology, is also interested in the question of the intentionality of consciousness. Keeping to the distinction between the pure consciousness and ego-consciousness, Advaita raises the question whether consciousness *per se* is intentional or whether the ego-consciousness is intentional. This question is important in the context of Husserl in as much as the distinction between “the pure I” and “the empirical I” is accepted by him. Is it the pure consciousness, “the phenomenological residuum,” that is intentional? Or, is it the *epoche*-performing ego that is intentional? This question need not be asked in the case of Sartre, because he not only holds that consciousness *per se* is intentional, but also accounts for the origin of the ego in terms of the intentionality of consciousness. Advaita maintains that consciousness *per se* is not intentional, but it becomes intentional because of the ego. Is there any evidence to say on the basis of a thoroughgoing application of the phenomenological method that consciousness is always and necessarily consciousness *of* something? Advaita answers this question in the negative.^16^ I shall argue this point on the basis of the phenomenological analysis as given in Advaita *Vedānta* which undoubtedly throws a new light on this problem.

It is unquestionably true that consciousness in our waking experience is always consciousness *of* something. In our waking experience, we do not have access to consciousness as such, apart from the object which it reveals and to which it is related. When we reflect on our consciousness, we know it to be intentional; we know it as the consciousness of this or that object. The intended object at this level may be physical like a table or a tree. Or, it may be a psychical state like pleasure or pain. In short, waking-consciousness is intentional. It must have transcendent objects related to it at this level. Being awake means being awake *to*. As in the case of waking experience, in dream experience also consciousness is intentional as it is always related to “objects.” My reflection on dream experience tells me that I was aware of many “objects” at that time.

As distinguished from waking and dream experience, there is the experience of sleep which is free from dreams. When a person wakes up from dreamless sleep and reflects on the nature of the experience he had, he says that at that time he was not conscious of anything whatsoever, objective or subjective. Nevertheless, there was consciousness at that time, though there were no objects, no phenomena, related to it. If consciousness were also absent at that time, recollection to the effect, “I was not conscious of anything then” would be impossible. The point is that consciousness reveals objects if they are present, and when there are no objects to be revealed, consciousness remains alone. It is, therefore, wrong to say that intentionality, as Sartre would put it, is consciousness, or that consciousness by its very nature is intentional. According to Advaita, consciousness becomes intentional only as a result of its association with the mind, and it has this connection with the mind in waking and dream experience. But in deep sleep experience, mind as mind is absent with the result that consciousness remains alone without being intentional. Advaita maintains that intentionality is not essential (*svābhāvika*), but only adventitious (*aupādhika*) to consciousness.^17^ Although it is true that there is no phenomenon without consciousness, there is no phenomenological evidence to say that there is no consciousness without the phenomenon.

On the basis of the distinction between consciousness and the ego, Advaita holds that not only the intentional act, but also the work of objectivation, identification, fulfilment and constitution mentioned by Husserl^18^ belong to the ego or the mind, which is transcendent to consciousness.^19^ It justifies this position on the ground that these cogitations are known in the same way as the external objects and their qualities are known, and that what is known must be transcendent to the knower. In other words, since consciousness is aware of these cogitations as they occur from time to time, as they appear and disappear in the mental horizon, they cannot belong to, or be part of, consciousness. For example, when someone sees an object, the object seen is transcendent to the observer. When someone perceives the whiteness, or the tallness, or the movement of an object, the quality or the action that is perceived cannot be the quality or the action of the perceiver, but must be the quality or the movement of the object in which it inheres. The same principle holds good in the case of the cogitations which one is aware of. These cogitations or mental operations too are objects of consciousness, but are not consciousness itself. It is relevant in this connection to refer to an Upani
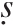
adic text which says: “Desire, resolve, doubt, faith, want of faith, steadiness, unsteadiness, shame, intelligence, fear-all these are but the mind.”^20^ What this text emphasises, though mentioning only a few of the mental operations in a suggestive way, is that all cogitations or mental operations are nothing but states of the mind and that they are not just because consciousness is aware of them, constitutive of consciousness.

It should be pointed out in this connection that what is commonly called the ego or the “I” is not consciousness, but the object of consciousness. If it were identical with, or part of consciousness, it could not be known by consciousness. We find that the ego or the “I” appears only during our waking and dream experience. In our waking experience, there is the “I” which functions as knower, (*jñātā*) as doer (*kartā*), as experiencer (*bhoktā*); it functions, that is to say, as the subject of knowledge, as the agent of action and as the experiencer of the consequences of action. In support of this, there is the evidence of the statements that we make from time to time with the first person singular such as “I know this,” “I do this,” “I reap the rewards of my actions” and so on. This is equally true in our dream experience which is very significantly on a par with our waking experience. The “I” is as much prominent in our dream experience as it is in our waking experience. A person perceives objects, performs deeds and suffers for his actions in dream experience, and is also aware of them. The dream world parallels the waking world, though there are also significant differences between them. The “I” which is present both in the waking and dream experience is absent in the state of deep sleep. Just as there is consciousness of the presence or absence of something, even so there is consciousness of the presence or absence of the ego or the “I” as the case may be. It follows that the ego or “I,” which is known as sometimes present and sometimes absent, is transcendent to consciousness.

If the ego or the “I” were identical with consciousness and not something transcendent to it, then what is it that is aware of it? Is it aware of itself? Or, is there anything else which could be aware of it? The first alternative is untenable, as it amounts to saying that one and the same entity is both the subject and the object at the same time in the same act of cognition. When we say that it is aware of itself, does it mean that this ego which is consciousness divides itself into two parts such that one part of it is the knower and the other part is the known? This is impossible, as consciousness is one and homogeneous and does not admit of division into two parts, viz. the subject-part and the object-part, according to Advaita. There is also another difficulty. If the ego, which is known, is identified with consciousness, then consciousness can never be the knower, the seer or the witness, to which everything is presented, that is, not being different from the ego, it becomes the known; if so, there will be no knower at all. It cannot be said, with a view to overcome the above difficulties, that consciousness which is the knower at one time becomes the known at another time. A thing is what it is, and it cannot become something different. It is impossible for a thing to change its essential nature, whatever may be the external form it may assume. Consciousness by its very nature is the seer all the time. And to say that it becomes the known is to assume a knower other than consciousness. And what is that which knows it? This question will now take us to the second alternative.

If the ego which is identical with consciousness, which is known by something else; that “something else” can not be the “object,” for an “object” is always what is known by, and what derives its meaning from, consciousness. On the contrary, that “something else” must be consciousness and not an object. It follows from this that one consciousness is known by another consciousness. If we persist in the same kind of questioning, we have to say that the second consciousness is known through a third one, and so on, leading to the fallacy of infinite regress. This difficulty apart, there is the unwarranted assumption that there is a plurality of consciousness. What is the evidence to show, Advaita asks, that there is more than one consciousness? To establish the existence of a plurality of consciousness, we require not only the differentiating features in terms of which we could say that one consciousness is different from another consciousness, but also a consciousness as the witnessing principle of these differentiating features. The features which help to distinguish one object from another are configuration (*avasthā*), place (*deśa*), time (*kāla*) and qualities (*gu*
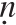
*a*). Two objects, we say, are different from each other because of their difference in configuration, their location in different places, their existence in different periods of time and the difference in their qualities. The question is whether differentiating features such as configuration, place, time and qualities can be associated with consciousness for the purpose of proving the existence of a plurality of consciousness. Every differentiating feature, it must be borne in mind, has to be noticed by consciousness. In that case, it becomes what is seen or witnessed (*sāk*
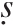
*ya*) by consciousness, and so it cannot belong to the latter. On the contrary, it must be transcendent to consciousness for the simple reason that it is seen or noticed by consciousness. It means that the existence of more than one consciousness cannot be proved. Therefore, the ego or the “I” which is transcendent to consciousness should not be identified with consciousness. There is nothing *in* consciousness, no content, no structures, no qualities and no parts, by which it can be identified and marked off from other things. All that can be said about consciousness is that it is revelatory of things presented to it, and it is by this nature that it is differentiated from the objects which it is aware of and which are, therefore, transcendent to it.

## Non-Egological Consciousness: Sartre and Śankara

It will be helpful in this connection to consider the non-egological theory of consciousness which Sartre formulates by placing the ego outside consciousness. According to Sartre, consciousness is non-substantial. Consciousness is “all lightness, all translucence.”^21^ It is not a container; it does not contain anything-no images, no representations and no contents. By its very nature, it transcends itself in order to reach an object and exhausts itself in this transcendence.^22^ When there is consciousness of a tree, the tree is not in consciousness-not even in the capacity of a representation.

In order to understand the impersonal nature of consciousness, it is necessary to start with the distinction introduced by Sartre between pre-reflective consciousness and reflective consciousness. The former is also referred to as a non-positional or non-thetic self-consciousness, whereas the latter is also called positional or thetic consciousness. The consciousness with which we start is the consciousness *of* something; it is the consciousness which is turned toward something other than itself. There is consciousness of a table, of a portrait and so on. Sartre maintains that consciousness of an object is at the same time consciousness of being conscious of an object. For example, when there is consciousness of a table, there is consciousness of being aware of the table. Consciousness not only reveals something, but also reveals itself. It means that at the time of the consciousness of the table, there is non-reflective awareness of consciousness. If this is not the case, it would be, Sartre argues, a consciousness which is ignorant of itself, that is to say, an unconscious being which is absurd.^23^

It is Sartre’s contention that the “I” or ego arises only at the reflective level. There is, first of all, let us say, consciousness of a tree. By reflecting subsequently on my intentional act and the intended object, I say, “I am conscious of the tree.” There is no place for the “I” or ego in the unreflected consciousness. Here is one of the examples given by Sartre, supposed to be based on the phenomenological analysis of the problem: “I was absorbed just now in my reading. I am going to try to remember the circumstances of my reading, my attitude, the lines I was reading. I am thus going to revive not only these external details but a certain depth of unreflected consciousness, since the objects could only have been perceived by that consciousness and since they remain relative to it. That consciousness must not be posited as object of a reflection. On the contrary, I must divert my attention to the revived objects, but without losing sight of the unreflected consciousness, by joining in a sort of conspiracy with it and by drawing up an inventory of its content in a non-positional manner.” There is no doubt about the result: while I was reading, there was consciousness of the heroes of the novel, but then I was not inhabiting this consciousness. It was only conscious of the object and non-positional consciousness of itself. I can now make these a-thetically apprehended results and object of a thesis and declare: there was no “I” in the unreflected consciousness.^24^ Just as there is no content in consciousness, even so there is no “I” or ego in it. Consciousness is, therefore, non-substantial and impersonal.

Sartre’s theory of non-egological consciousness is acceptable to the Advaitin. Sartre’s explanation of consciousness as impersonal and non-substantial, as “a great emptiness,” is a reiteration of the Advaita view that consciousness is not a substance possessing attributes, that it is not a whole consisting of parts, that it is not an entity which can be specified as such-and-such, as it is free from class feature, qualities, action, and relation.^25^ The view that the ego or “I” which is transcendent to consciousness arises only at the reflective level of consciousness is the echo of the Advaita view which holds that the ego (*aham*) arises when there is *cidābhāsa*, that is, consciousness reflected in the internal organ,^26^ and this consciousness associated with the internal organ, which alone is capable of reflection, may be characterised in the terminology of Sartre as consciousness in the second degree. Sartre does not deny the existence of the ego, but only denies that it is in consciousness. He says that the ego which is transcendent to consciousness is the unity of subjective states and actions known through reflection. There is, Sartre observes, something mysterious, irrational about the ego.^27^ We cannot apprehend the ego apart from states and actions. If we take away one by one all the states and actions, the ego would disappear. According to Sartre, spontaneity is what characterises consciousness. If the ego appears to have spontaneity, it is because consciousness projects its spontaneity into the ego. This account of the ego as a mystery and a problem, as that which functions through the borrowed light (spontaneity) of consciousness, is fully amplified in all the major works of Advaita.^28^ The difficulty arises only when Sartre denies the role of the transcendental consciousness as the unifying principle of the intentional acts. Sartre argues that the transcendent object intended by consciousness gives unity to the different intentional acts. His argument is not convincing. It appears that Sartre who banishes the transcendental “I,” the permanent factor underlying all our acts of consciousness, by the front door brings it in through the back door. At every stage in his explanation, he tacitly assumes the existence of such a permanent consciousness in all our acts of consciousness. Sartre thinks of consciousness as something individualised and particularised by the objects to which it is related. He also thinks in terms of a flux of consciousness.^29^ Very often, he uses the expression, “fleeting consciousness.”^30^ Though there are innumerable acts of consciousness (i.e., intentional consciousness) coming one after another in a regular procession, whether they are related to one object or different objects, it is quite possible, according to Sartre, to connect all of them in reflection, as if there were a common identical factor running through them. I shall focus attention on two examples given by Sartre.

We have already said that transcendental consciousness is renamed empirical consciousness when it is in the embodied condition. With the empirical dress, it puts on followed by its involvement in the world; it is called the enworlded subjectivity. The point to be noted here is that absolute consciousness is immanent in its empirical appearance, and performs all cognitive functions, retaining its essential nature. That is because it is also at the same time transcendent to it. The following diagram brings out the immanent -transcendent dimension of the absolue consciousness [seeFigure 1].

**Figure 1:**
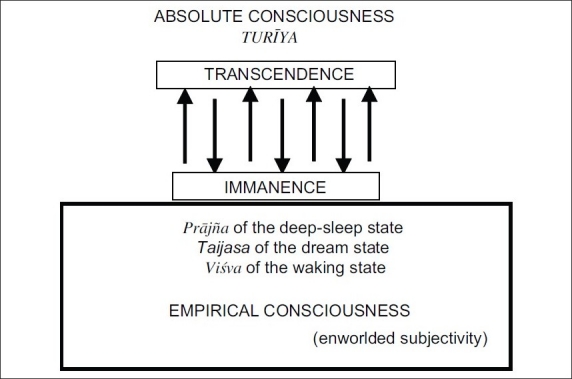
Immanent-transcendent dimension of absolute consciousness

Though empirical consciousness at every stage of its functioning reveals what it is and how it is related to the objects which are transcendent to it, it does not show the *absoluteness* of its real nature. As we are absorbed in the things of the world revealed to us by the consciousness, we do not normally probe into its real nature. Even though it is the source of the world, it remains concealed in the latter in the same way as clay, which is the source or ground of the pot made out of it, remains concealed therein. The manifestation of the pot is the concealment of clay, which is its cause; and the revelation of clay is the concealment of the pot. There is thus the mysterious play of hiding and showing by consciousness. What is true of clay and pot in this example is true of absolute and empirical consciousness.

Now, the important question we have to consider is whether it is correct to characterise absolute consciousness as both transcendent and immanent. The answer is both yes and no. We claim that we are rooted in the world, subjective and objective. The mind, the senses and the body which we possess, we claim, are real, and so is the external world of space, time and causality. It is but natural for us to begin to think of the cause of the world and search for it as we are already deeply entrenched in the world accepting its reality. Our philosophical reflection on this problem is the first attempt to overcome the naive and natural attitude toward the world, both subjective and objective. Again, as we try to know the truth of the mind-sense-body world through a process of transcendence from one level to another-from the bodily to the vital, from the vital to the sensory and then to the mental and the intellectual, and finally to the self-consciousness-we will discover the Self or consciousness which remains hidden, supporting the entire mind-sense-body complex, and which is the transcendental *a priori* of all that we do as the subject of knowledge, as the agent of action and the enjoyer of the consequences of our action. Being of the nature of consciousness, the Self, though immanent in the mind-sense-body complex, is not only different from it, but is also transcendent to it. Similarly, if we probe into the external world by subjecting the things therein to a rigorous causal inquiry, then we can *discover* the primal being, which the (*Upani*
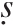
*ads*) call Brahman/*Ātman*, which is absolute consciousness, as not only the final cause of the world, but also its ground. What is identified as the final cause is really its ground. Brahman/*Ātman*, as the cause of the world, is immanent in it, and so from the perspective of the world, the theory of the immanence of Brahman/*Ātman* is justified. The concept of “*tajjalān*” which is formulated in the (*Upani*
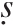
*ad*) in the process of the causal inquiry also shows that what really exists is Brahman/Ātman and that the entire manifested world which has a dependent origination and existence is kalpita. If so, Brahman/*Ātman*, the absolute consciousness, which is real, is other than the world, and is transcendent to it. It means that the concept of the transcendence of Brahman/*Ātman*, or the absolute consciousness, is intelligible only on the presupposition of the existence of the world. It must be borne in mind that Brahman and Ātman are one and the same entity. The two terms, “Brahman” and “Ātman,” have the same referent. In the absence of the world, there is neither the immanence nor the transcendence of Brahman/Ātman, though for the purpose of instruction (*upadeśa*), the *Upani*
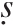
*ad* speaks of it as immanent as well as transcendent. *Gaudapāda’s* declaration, “*upadeād-aya*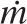 *vāda*
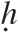
” and “jñāte dvaita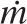 *na vidyate,”*^31^ is relevant in this context.

There are two questions to be considered in this connection. First, even though the absolute consciousness, the Fourth (*turīya*) as it is called in the context of the triple stream of experience, is beyond the grasp of the senses and the mind, why is it that it cannot be spoken about? Second, if it is, as stated earlier, unperceivable and unseen, uninferable, beyond thought and beyond empirical dealings, then how is it known? Both the questions are important, and we will consider them one by one.

There is a fundamental distinction between the empirical and the transempirical. Any object which is empirical is a limited entity, and whatever is limited is necessarily relational. An object which is empirical and therefore relational will have class feature (*jāti*), quality (*gu*
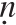
*a*), action (*kriyā*) or relation (*sambandha*), or it may be signified by a conventional word (*rū*
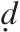
*hi*) used only with reference to it. For example, an object which possesses the class feature, viz. cowness, is signified by the word “*go*.” An object which possesses the quality, viz. white colour, is spoken of as “*śukla*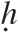. ” Similarly, we call the cook a “*pācaka*
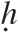
” as he performs the act of cooking. One who possesses wealth, that is, one who has relation with wealth, is called a “dhanī.”. An object which provides space is conventionally called “*ākāśa*.” So, there are reasons such as the class feature, quality, action, relation and conventional usage for the application of words to objects. Since none of these features are present in the transempirical Self, it cannot be directly signified by a word. It is for this reason that *śruti* says that the Self is “that from which speech returns.”^32^ However, it can be secondarily signified, according to Sureśvara, by the words “I” and “thou,” because the primary sense of these words is the knower (*pramātā*), and the Self, being its witness, is connected with it. A note of caution is necessary at this stage. We resort to the secondary sense when the primary sense does not hold good, or does not convey the intended meaning of a sentence, oral or written. However useful the negative scriptural texts like “*neti neti*” may be, still they have their own limitations. Though they tell us what the ultimate reality is not by denying every predication that we make, they do not and cannot tell us what its real nature is. One may urge the same argument against the adoption of the secondary sense for construing the meaning of a text about the ultimate reality. Since an empirical object falls within the scope of language, there is justification for adopting the secondary sense in lieu of the primary sense when the context needs it. The use of pronouns such as “I” and “thou” is restricted to the empirical realm of ordinary discourse. The functional consciousness falls within the scope of ordinary empirical discourse, and so it may be signified by pronouns such as “I” and “thou” as required by the context. But there is no scope for *śabda*, both secular and sacred, in respect of the ultimate reality which is transempirical and transrelational. The (*Upani*
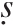
*adic*) declaration that speech returns without reaching the Ultimate restricts the scope of language to the empirical, and does not admit of any compromise in its operational scope. “The boundaries of my language,” says Wittgenstein, “are the boundaries of my world.” Then he goes on to say: “What we cannot think, we cannot think; and we cannot *say* what we cannot think,” and “What one cannot speak about, one must pass over in silence.”^33^

We will now take up the second question. The Self or consciousness, being self-luminous, is self-established. A brief explanation will be helpful to understand this point. It is through consciousness that everything, whether it is an object in the external world, or one’s own body, whether it is a mental state like pleasure or pain, or mind itself, is known. By itself, the internal organ (*anta*
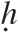
*kara*
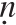
*a*) which is material cannot cognise or reveal anything, much less consciousness on which it is dependent. If it gets the status of a knower (*jñātā*), it is because of the fact that the foundational consciousness, that is, the Self, is reflected therein. The internal organ, carrying the reflection of consciousness, knows itself as “I” (*aham*). In the same way, it knows other objects which are presented to it as “this” (*idam*). Starting from the internal organ which is material and which is other than the Self or consciousness, every object is known only through consciousness. Furthermore, the internal organ is never constant. It is subject to modifications. The mental modes, that is, the changes of the internal organ, which appear and disappear one after another, are known only through the Witness-consciousness (*sāksi-caitanya*), which alone is eternal and self-luminous.^34^ The reason for this is obvious. The mental modes form a series, and a series can never be aware of itself as a series, but can be known only through another factor outside it, which is both permanent and a witness to it.

It is of no use to invoke the help of a *pramā*
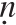
*a* in this regard. A *pramā*
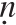
*a* can function as a *pramā*
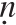
*a* and generate knowledge of anything only through the help of consciousness. We can go one step further. The very distinction between *pramā*
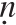
*a* and a *pramā*
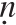
*a* presupposes the work of consciousness. If we say that something is a *pramā*
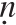
*a* and that something else is not a pramāna, it is because of the Witness-consciousness. In the same way, it is only through the Witness-consciousness that we are able to distinguish a valid cognition (*pramā*) from an erroneous one (*ābhāsa-jñāna*). Consciousness which is presupposed in all acts of knowing is the basis of all knowledge. Although other objects are established through consciousness, the latter is self-established (*svatassiddha*), for it is self-luminous by its very nature. Consciousness is self-luminous in the sense that although it is not revealed by any other means or agency, it reveals other objects. That is why it is said to be the transcendental *a priori*. That which is presupposed by all *pramā*
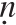
*as* and all acts of cognition cannot be proved by them, and it does not require any proof. It is as good as proved.^35^

## The Popperian Model of Three Worlds

**Figure 2:**
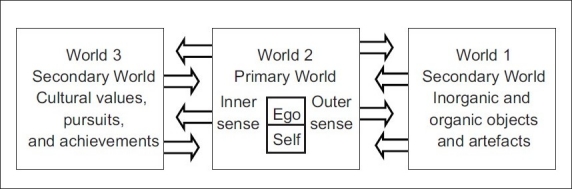
The three worlds model of Popper

The Popperian theory of three worlds, which has been adopted by John Eccles, is substantially the same as the *Upani*
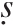
*adic* theory of three worlds. Assigning the central place to the pure Ego or the Self in the life-world of the human person, Karl Popper and John Eccles speak of World 1, World 2 and World 3. Of these, World 2 which is characterised as the world of consciousness is designated the primary reality, whereas World 1, which is the external world of matter and energy, and World 3 which is the world of culture are given the status of secondary realities. The following tabular form, which is adapted from the one given by Eccles, gives a picture of the three worlds and the interaction among them [see Figure 2].^36^

In the above classification, World 1 comprises the entire cosmos of matter and energy, the biological structure and actions of all living beings including human brains, and the artefacts that are creations of human beings. It is the external world of space, time and causality. The interaction between the *jīva* and the external world is through the outer senses and the brain. According to Eccles, the brain which is viewed as a part of World 1 provides the communication line between World 1 and World 2. It is, therefore, characterised as the “liaison brain.” Since the interaction between the self-conscious mind and the external world takes place through the brain, Eccles holds that the brain is “necessary, but not sufficient for World 2 existence and experience.”^37^ There are three segments in World 2. All our sensations of sound, heat and cold, colour and light, taste and smell belong to the area of the outer senses, whereas our thoughts and memories, feelings and imaginations, intentions and volitions constitute the inner level. The pure Ego or the Self is in the centre stage, supporting, controlling and unifying the functioning of the outer senses and the inner mind. Just as the *Upani*
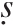
*adic* say that the Self or the foundational consciousness is the invariable factor in all our experience connecting one state of experience with another,^38^ even so Popper and Eccles speak of the Self or the Ego as the bridge connecting the different states of experience. To explain the functioning of the Self in the day-to-day life of a human person, Eccles quotes the following passage from Sherrington:

Each waking day is a stage dominated for good or ill, in comedy, farce or tragedy, by a dramatis persona, the “Self”. And so it will be until the curtain drops. This Self is a unity. The continuity of its presence in time, sometimes hardly broken by sleep, its inalienable “interiority” in (sensual) space, its consistency of viewpoint, the privacy of its experience, combine to give it status as a unique existence.^39^

It may be noted that in the Popperian analysis of the conscious experiences of World 2, the subtle distinction between the Self and the mind, or foundational consciousness and functional consciousness that has been highlighted earlier during the explanation of the triple stream of experience does not emerge. However, both Popper and Eccles speak about mental phenomena or subjective states of which a human being is conscious. The expression “self-conscious mind” occurs frequently in the writings of Eccles. If we follow the *Upani*
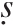
*adic* tradition, it has to be emphasised here that even though there is the mind-brain interaction, the mind is as much material as the human brain which is admitted to be a part of World 1. In fact, according to the *Upani*
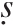
*adic* tradition, everything in the human being excepting the Self or consciousness is material (*ja*
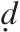
*a*).^40^ So, if the mind which is material becomes self-conscious, it must be due to the presence of an extraneous factor which helps it to become self-conscious, to develop an awareness of itself. It may be added that the mind which is the beneficiary in this process must be dependent on this extraneous factor which is its benefactor. We have already stated that the proximity of the mind to the Self makes it an instrument of cognition as a result of the reflection of the power of consciousness of the Self in it. Carrying the reflection (*pratibimba*) or semblance (*ābhāsa*) of consciousness, the mind becomes a sentient entity as it were, is endowed with the power of cognition of other objects, develops the sense of “I” and “mine” and also becomes self-conscious when the need arises. Consider the following functions of the self-conscious mind as enumerated and explained by Eccles on the basis of the hypothesis of a strong dualism:^41^

1. The self-conscious mind is an independent entity, a World 2 existence, which has a status in reality equivalent to that of the brain with its World 1 existence.
2. It acts upon the neural centres modifying the dynamic spatiotemporal patterns of the neural events. It means that the self-conscious mind exercises a superior interpretative and controlling role upon the neural events.
3. It alone provides the unity of conscious experience and not the neural machinery of the liaison areas of the cerebral hemisphere.

The work that the self-conscious mind does is marvellous. It elevates the status of the *jīva* from the animal level to that of a human person. What is called the “*self-conscious mind*” is not a simple entity, but a complex of three factors, according to Advaita. It is a blend of the mind, the foundational consciousness that supports the mind and the reflection of the consciousness in the mind.^42^ It has, therefore, to be distinguished from the Self or foundational consciousness. It means that there are in the present context two different categories, viz. the Self and the mind, which should not be mixed up with each other. Eccles seems to be aware of the distinction between the pure Ego and mental phenomena or conscious experiences which would include all mental episodes including self-consciousness, and this is evident from his statement that although the conscious experiences are perceived by the Ego or the Self, the latter is experienced, not perceived. In support of this, he quotes Polten who, following Kant, draws the distinction between them:

The ontological basis for the difference between apperception and perception is that the pure Ego is a mental thing-in-itself, whereas the mental phenomena of inner and outer sense are appearances. For that reason, too, subject and object merge in the act of the pure ego’s self-observation, while inner and outer data are the pure ego’s objects.^43^

The epistemological distinction between perception and apperception points to the ontological distinction between the mental phenomena and the mental thing-in-itself, and if the mental phenomena are appearances, then the mental thing-in-itself must be the reality. The latter is what the Advaitin would call the Self or the foundational consciousness.^44^

Although the human being is moulded and shaped by the world of culture, the latter in its turn is shaped and sustained by the human beings. The producer of culture is at the same time the product of his culture. It means that they influence each other. The transition from a human being to a human person is due to the development of self-consciousness, which is facilitated by the world of culture. Eccles gives a graphic picture of the contents of the world of culture after raising the question, “What is World 3?”

It is the whole world of culture… World 3 was created by man and that reciprocally made man. The whole of language is here. All our means of communication, all our intellectual efforts coded in books, coded in every artistic and technological treasure in the museums, coded in every artefact left by man from primitive times-this is World 3 right up to the present time. It is the world of civilization and culture. Education is the means whereby each human being is brought into relation with World 3. In this manner, he becomes immersed in it throughout life, participating in the heritage of mankind and so becoming fully human. World 3 is the world that uniquely relates to man. It is completely unknown to animals.^45^

He goes on to say:

This World 3 provides the means whereby man’s creative efforts live on as a heritage for all future, building the magnificent cultures and civilisations recorded in human history.^46^ It may be noted in this connection that the social dimension of Advaita emphasises the importance of the tradition of society in shaping the life of an individual. Śa
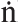
kara points out that every human being has two-fold competence-eligibility for the pursuit of knowledge and eligibility for the performance of willed action (*jñāna-karma-adhikāra*).^47^ The two-fold competence helps an individual not only to inherit the value system of the society, but also to transmit it to posterity.

## Is Knowing without Mental Operation Possible?

The distinction between foundational consciousness and functional consciousness is required for the purpose of epistemological and metaphysical analysis. Since consciousness is one and only one, it may appear that there is no justification for such a distinction. However, the need for such a distinction arises because our philosophising starts from the given world, the world of our everyday experience, and consciousness which is involved in, or associated with, the objects of the world, is characterised by its worldliness. Its relation with the objects is two-fold; on one hand, it is involved in the mind-sense-body complex of the individual, such that it becomes embodied consciousness; on the other hand, it is also associated with the objects of the world as the principle responsible for the manifestation and meaning of the entire world. The concept of “constitution” plays an important part in Husserlian phenomenology. According to Husserl, we have to understand the sense and being of the objects only in terms of the work of constitution by consciousness. He explains “constitution” sometimes as sense-bestowing, sometimes as “producing,” as “making,” as “creating” and so on.^48^ There is no need to go into the details about the work of constitution by consciousness. However, the point to be noted here is that the kind of objectivity which the things of the world have is bestowed on them by consciousness, and the object is unthinkable apart from consciousness. In the words of Dermot Moran:

Constitution is a universal feature of conscious life; all meanings are constituted in and by consciousness. Everything experienceable in both the natural and cultural world is constituted, as Husserl argues in Ideas II…. Husserl speaks of the living body constituted by its kinaesthetic functions…. also, he talks of the constitution of social and cultural entities. This last is more familiar, particularly since, throughout the twentieth century, there has been much talk of the “socially constructed” nature of social entities such as families, institutions, banks, money, and so on. In this sense, constitution can be considered as similar to social construction. However, Husserl goes much further than social constructionists in that, for him, even things of nature are constituted.^49^

Following the *Upani*
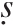
*ads*, Advaita holds that consciousness is the support (*adhi*
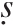

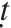
*hāna*) of the objects of the entire world; that is to say, the objects, which are totally different from consciousness, have no existence of their own, no status of their own, no nature of their own, with the result that they are dependent on consciousness.^50^ What is inexplicable is that consciousness which has no relation with anything-for there is no other entity which can be reckoned as real to come into relation with-comes to be related with the objective phenomena. There is no objective world which exists independently of consciousness, and what appears as the objective world conditioned by space, time and causality is the manifestation of the foundational consciousness. Advaita holds that consciousness is the transcendental *a priori* of all objects, both for the purpose of their existence and knowing.^51^ The point to be noted here is that Advaita does not deny the existence of the objective world, what it denies is the independent existence of the objective world.^52^ The standpoint of the Husserlian phenomenology is surprisingly the same. In the words of Aron Gurwitsch:

From the phenomenological point of view, consciousness cannot be regarded as one mundane realm among others. To whatever mundane realm an object belongs, it necessarily involves implicates, and in this sense, presupposes consciousness, namely, those acts through which the object in question appears and displays itself as that which it represents in our life. Consciousness thus reveals itself as the universal domain or medium of presentation of all objects, a domain to which every mundane realm necessarily refers. Herein consists the privilege and the priority of consciousness to every mundane realm. The mundane nature of a realm purports its insertion as a part into the whole of the total reality. In this sense, mundane nature must not be ascribed to consciousness.^53^

## Concluding Remarks [see also Figure 3]

**Figure 3:**
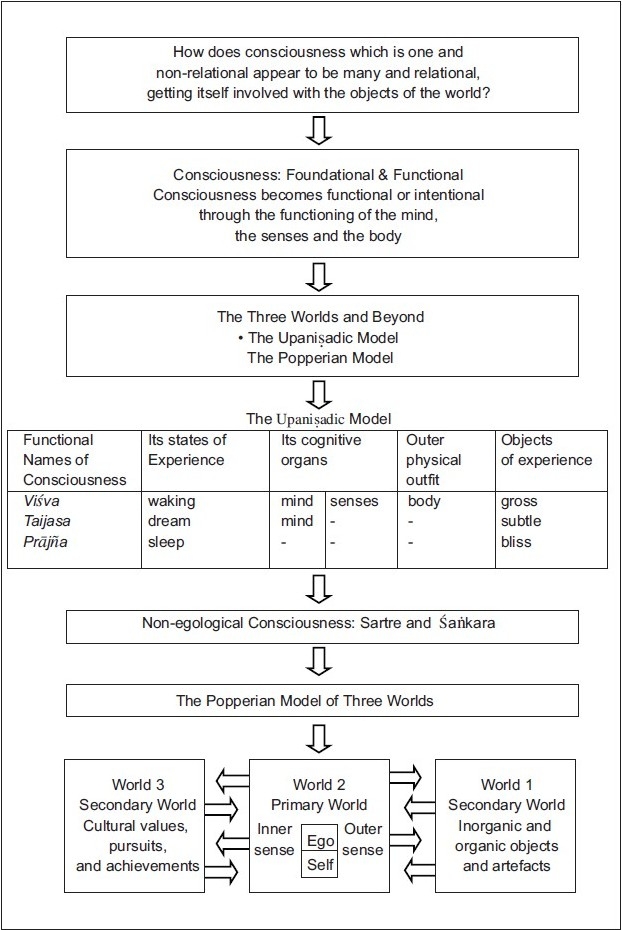
Flow chart of the paper

Advaita *Vedānta* may be characterised as “transcendental phenomenology” and “metaphysics of experience.” Adopting the phenomenological method, it holds that the evidence of consciousness is the only one that is certain and apodictic for any claim that we make–claim that we know something as well as the claim that we do not know something. Every source of knowledge, be it perception or inference or scriptural testimony, is dependent on consciousness, which is not only self-luminous, but is also revelatory of our objects of experience. That which is the presupposition of every kind of knowledge and of every source of knowledge cannot be validated by any other principle.

Consciousness which is non-functional by its very nature becomes functional through its association with the mind-sense-body complex, and has three levels of experience–waking, dream and deep-sleep. It holds that every object of experience is real and that there is a hierarchy of reality of objects, and so it moves from empirical pluralism to transcendental monism.

## Take home message

Any system of philosophy can be correctly understood and appreciated only if it is viewed through the right perspective. The distinction between the absolute and the relative points of view runs through the entire system of Advaita. What is true from one point of view or at one level is not true from another point of view or at another level. Though Advaita is pluralistic from one point of view, it is monistic from another point of view. Without denying pluralism, it affirms monism.

## Questions that this Paper Raises

1. Is consciousness by its very nature intentional?
2. How does one know one’s mind?
3. Where is the mind located? How do the mind and the brain interact?
4. Are there innate ideas in the mind? Are they predetermined? If so, how?
5. What is the solution to the problem of “freewill vs. determinism”?

## About the Author

*Prof R. Balasubramanian is a specialist in Advaita, Phenomenology and Existentialism. He is at present Hony. Visiting Professor, Sri Aurobindo School of Eastern and Western Thought, Pondicherry University and President, Afro-Asian Philosophy Association. He was Director, Radhakrishnan Institute for Advanced Study in Philosophy, University of Madras, for many years. He was also Chairman of Indian Council of Philosophical Research. He has a number of books and papers to his credit. Some of his publications include Personalistic Existentialism of Berdyaev (1970),The Taittiriyopanisad-bhasya-vartika of Suresvara (1974, 1984), A Study of the Brahmasiddhi of Mandanamisra (1983) and The Tradition of Advaita (editor) (1994).*
